# Cardiac interoception in chronic pain: no association between interoceptive accuracy and symptom report

**DOI:** 10.3389/fpsyt.2026.1813993

**Published:** 2026-09-09

**Authors:** Mirjam V. Thomas, André Schulz, Dimitri M. L. van Ryckeghem, Annika P. C. Lutz, Kai Lambracht, Jens Adermann, Claus Vögele

**Affiliations:** 1 Department of Behavioural and Cognitive Sciences, University of Luxembourg, Esch-sur-Alzette, Luxembourg; 2 Department of Clinical Psychological Science, Maastricht University, Maastricht, Netherlands; 3 Department of Experimental Clinical and Health Psychology, Ghent University, Ghent, Belgium; 4 Klinik für Manuelle Therapie Hamm, Hamm, Germany

**Keywords:** chronic pain, heart-beat evoked potentials, interoception, interoceptive accuracy, perception filter model

## Abstract

**Background:**

The etiology of many chronic pain conditions is still poorly understood. According to the perception-filter model, amplification (stage I.) and insufficient filtering (stage II.) of afferent bodily signals results in the increased perception of physical sensations (stage III.).

**Methods:**

Focusing on cardiac interoception, we tested if these hypothesized alterations in stages I.-III. apply to a sample of 76 in-patients with chronic pain (M_age_ = 50.7, 53 females). Heart rate (HR) and heart rate variability (HRV) were assessed as indicators of afferent bodily signal strength (stage I.). The difference between heartbeat-evoked potentials (HEPs) during a resting phase and a heartbeat counting task (HCT), i.e., ΔHEP, was used as an indicator of filter system activity. Interoceptive accuracy (IAcc) in the HCT and a heartbeat discrimination task (HDT) served as indicators of perception of afferent bodily signals.

**Results:**

HR did not predict either indicator of IAcc. LF HRV was a positive predictor of IAcc_HCT_ and HF HRV positively predicted both IAcc_HCT_ and IAcc_HDT_. HEP amplitudes did not differ between the HCT and the resting period and ΔHEP did not moderate any of the relationships between indicators of bodily signal strength (stage I.) and IAcc (stage III.). Neither indicator of IAcc (stage lll.) was significantly associated with measures of symptom report.

**Conclusion:**

Alterations in the processing of bodily signals as suggested by the perception-filter model do not apply to chronic pain conditions. Future studies should focus on investigating pain-specific processing of bodily signals.

## 1 Introduction

Chronic pain has a detrimental impact on patients’ quality of life (1), is associated with poor mental health (2), and puts a significant strain on society by reducing occupational functioning (3) and resulting in high healthcare costs (4). Despite an estimated worldwide prevalence of 20% (5, 6) and repeated calls from the scientific community to consider chronic pain as a global health priority (7), the genesis of many chronic pain conditions is still poorly understood (8). As chronic pain often lacks a linear association between identifiable pathophysiology and pain (9), traditional biomedical models largely fail to explain its etiology. Instead, a biopsychosocial model, in which pain is assumed to result from a complex interplay of various biological, psychological and social factors, is assumed to be the most comprehensive approach for understanding persistent pain (10). To improve the prevention and treatment of chronic pain, in-depth knowledge about the mechanisms driving its pathology is of crucial importance.

One of these mechanisms concerns interoception, i.e., the processing and perception of afferent internal bodily signals (11). In previous research alterations in the accuracy of perceiving internal physical signals (interoceptive accuracy: IAcc) (12) have mostly been associated with mental disorders. These results suggest that interoceptive alterations may be a transdiagnostic factor across several mental disorders. More recently, it has been hypothesized that interoception plays a similar key role in the development and maintenance of chronic pain (13, 14). This hypothesis is supported by neurobiological findings indicating that the processing of pain and the processing of viscerosensory information engage common neural substrates such as the insular cortex, the anterior cingulate cortex, and the somatosensory cortex (15, 16). Moreover, different models aiming to explain chronic somatic symptom distress like persistent pain emphasize the role of interoceptive alterations in the generation and perpetuation of physical symptoms (17). These models share the assumption that the perception of benign bodily signals constitutes the starting point of a vicious circle of dysfunctional attentional (e.g., selective attention), cognitive (e.g., catastrophizing) and behavioral (e.g., scanning the body for signs of a disease) processes, which leads to the development of somatic symptoms such as chronic pain (17). Most of these models do not specify potential underlying physiological mechanisms. The perception-filter model, however, focuses on neurophysiological states contributing to the perception of physical symptoms (18, 19). It assumes that the brain continuously receives sensory input from the periphery of the body (stage I.). To avoid over-stimulation, most of these signals are, however, filtered by the nervous system (stage II.) and therefore only relevant information reaches higher cortical structures and is perceived (stage III.). According to this model, somatic complaints such as chronic pain develop as a result of distortions at all three stages of the processing of sensory input: (I.) afferent bodily signals are amplified due to autonomic over-arousal or chronic activation of the hypothalamic-pituitary-adrenocortical (HPA) axis; (II.) sensory input is insufficiently filtered as a result of decreased filter capacity because of selective attention or anxiety; (III.) this results in the heightened perception of physical signals and symptoms, i.e., pain.

Although highly relevant for advancing the understanding of chronic pain, the empirical evidence regarding interoception in chronic pain is scarce (14). This study aimed at further investigating the role of interception for chronic pain by examining the relationships between cardiac indices, interoceptive accuracy, and symptom report in chronic pain. The theoretical background was provided by the perception-filter model. So far, hypotheses derived from the perception-filter model have been mostly tested in individuals with medically-unexplained symptoms (MUS) (20, 21), that is, persons experiencing persisting heterogeneous somatic symptoms without sufficient medical explanation. In patients with chronic pain conditions, however, most of previous research examined different elements of the model in isolation (19). Such isolated assessment of elements of the perception-filter model limits the comprehension of underlying mechanisms behind the processing of bodily signals. Thus, the aim of the current study was to investigate potential alterations of the processing of bodily signals in chronic pain. To address this aim, we focused on the three stages (I.: afferent signals, II.: filter system, and III.: physical symptoms) as suggested by the perception-filer model.

We focused on the processing and perception of cardiac signals, i.e., cardiac interoception, as to date only the cardiac domain allows for the assessment of indices for all three stages of the perception-filter model. Higher heart rate (HR) and low frequency heart rate variability (LF HRV) are sensitive to central sympathetic activation (22, 23), whereas high frequency (HF) HRV is typically seen as index of central parasympathetic tone (24). As sympathetic and parasympathetic tone affect key properties of cardiac signals, such as chronotropy or stroke volume, and therefore inotropy, afferent bodily signal strength (stage I.) was estimated using HR and HRV (25, 26). According to previous studies (20, 21), the operationalization of the filter system activity (stage II.) was based on heartbeat-evoked potentials (HEPs), that is, electrocortical potentials related to the processing of cardiac signals (27). The amplitude of the HEP increases when attention is allocated to the heartbeat (28). Hence, the difference between HEPs assessed during a heartbeat counting task (HCT) (29) and a resting period (i.e., ΔHEP) reflects the differentiation between the signal (i.e. heartbeat) and the background noise. As this differentiation can be construed as filter of cardiac signal processing (20, 21), ΔHEP was used as an indicator of filter system activity. IAcc in the HCT and a heartbeat discrimination task (HDT) (30) served as an indicator of perception of afferent bodily signals (stage III.).

The perception-filter model posits that MUS are characterized by (I.) increased bodily signals, (II.) a deficient filter system and (III.) increased symptom perception. Furthermore, it has been assumed that these stages are inter-related (18–21). As many MUS involve pain symptoms, MUS and chronic pain may share important pathological mechanisms. In line with the model, we expected (1.) parameters of sympathetic arousal to be positive and parameters of parasympathetic arousal to be negative predictors of IAcc. We further hypothesized that the strength of filter system activity would (2.) moderate the association between parameters of sympathetic arousal and IAcc in a way that higher filter system activity as indicated by higher ΔHEP would weaken the positive relationship between parameters of sympathetic arousal and IAcc. Additionally, we predicted (3.) IAcc to be positively associated with symptom report, i.e., intensity of pain.

## 2 Material and methods

### 2.1 Participants

In this cross-sectional, experimental study, patients receiving inpatient interdisciplinary multimodal pain therapy at a tertiary pain hospital in Germany were recruited for participation between November 2020 and August 2021. Patients were eligible to participate if they were between 18 and 65 years old, reported pain that lasted or continuously reoccurred for at least 3 months, and had sufficient knowledge of the German language. The following exclusion criteria were applied: (1) history of major neurological disorders, (2) current or past diagnosis of a psychotic disorder, (3) uncorrected impairment of vision or hearing, (4) current intake of benzodiazepines, (5) regular consumption of alcohol or illicit drugs, (6) conditions impeding the application of EEG (e.g., allergies), (7) proneness to experience severe discomfort in medical settings (e.g., fainting), (8) proneness to experience severe discomfort when focusing on the heartbeat and (9) inability to use both index fingers. The latter exclusion criterion pertained to a task, which was part of the extended study setup that will be reported elsewhere. Patients were informed about the study in a brief face-to-face meeting on the second day of their inpatient stay at the hospital. In case they indicated interest in participating in the study, inclusion and exclusion criteria were checked and written information about the study was provided. Of the 158 patients who were informed about the study, 40 individuals were not interested in participating and 23 did not meet the eligibility criteria of the study. The main reasons for exclusion were regular use of alcohol or illicit drugs (*n* = 10), a history of major neurological disorders (*n* = 6) and insufficient knowledge of the German language (*n* = 2). As a result of delayed access to the final medical records, three more patients were identified as not meeting the eligibility criteria due to a history of major neurological disorders after participating in the study and were thus excluded from further analyses. Additionally, five patients withdrew their consent to participate in the study, and seven patients discontinued study participation after the first session of the study due to an acute exacerbation of their pain condition (*n* = 5), severe opioid withdrawal symptoms (*n* = 1) and treatment dropout (*n* = 1). Two patients had to be excluded from analyses due to excessive artifacts in the EEG data and another two patients were excluded due to underlying cardiovascular diseases which rendered the analysis of the ECG data within the scope of this study impossible. Therefore, the final sample comprised 76 patients.

### 2.2 Procedure

The study was approved by the Ethics Review Panel of the University of Luxembourg (ERP 20-022A). All participants provided written informed consent. Study participation was split over two sessions, which took place on separate days during the patients’ stay at the hospital. In the first session, a brief custom-made interview was conducted, and participants completed a questionnaire. This was followed by a computerized task assessing interpretation bias of ambiguous situations (results will be reported elsewhere). In the second session, psychophysiological monitoring was employed. After electrode application and a rating of their current pain intensity, patients were seated in front of a computer (Dell Latitude E5540). The assessment started with a 5-minute resting period during which participants kept their eyes open and closed for 2.5 minutes each. The order of closed and open eyes was counterbalanced across patients. Participants then completed the HCT and subsequently the HDT. The session ended with a dot-probe task measuring attention bias toward pain-related information (results will be reported elsewhere). The E-Prime 3.0 software (Psychology Software Tools, Pittsburgh, PA) was used to present tasks and collect responses. Additionally, participants self-administered several questionnaires both after the brief recruitment meeting and the first session of the study.

### 2.3 Medical record and self-report data

Data on current medication and diagnoses according to the International Statistical Classification of Diseases and Related Health Problems, 10th revision (ICD-10) (31) were extracted from the medical records of the patients available after their discharge from the hospital. Demographic information and data on height, weight, duration of pain and pain location(s) were collected via the custom-made interview.

Severity of chronic pain in the past three months was assessed with the German version of the Chronic Pain Grade (CPG) (32). The CPG comprises 7 items and yields a total score which allows to classify the severity of chronic pain as (1) Grade I: low disability and low pain intensity, (2) Grade II: low disability and high pain intensity, (3) Grade III: high disability and moderately limiting, and (4) Grade IV: high disability and severely limiting. Except for the item assessing the number of disability days, all items are rated on an 11-point rating scale (range 0 to 10), where higher scores reflect greater pain intensity respectively disability. The CPG was administered in the first session of the study. Current pain intensity at session 2 was assessed with the first item of the CPG. The psychometric properties of the German CPG are well established (32).

Pain intensity, depressive symptoms and anxiety in the past seven days were assessed using the pain intensity scale and the depressive symptoms and anxiety domains of the PROMIS-29 v2.1 Profile (33), a generic health measure. While pain intensity is assessed with a single item on a 11-point Likert scale (0 = no pain to 10 = worst imaginable pain), the depressive symptoms and anxiety scales consist of four items each and are scored utilizing a T-score metric where higher scores indicate more depressive respectively anxiety symptoms (34). The HealthMeasures Scoring Service (https://www.assessmentcenter.net/ac_scoringservice) was used to compute standardized T-Scores with a mean of 50 and a standard deviation of 10 (35). The PROMIS-29 v2.1 Profile has shown good psychometric properties (35).

### 2.4 Heartbeat perception tasks

The HCT (29) comprised six experimental intervals (25 s, 35 s, 45 s, 55 s, 65 s, 75 s), which were presented in randomized order. Patients were instructed to count all of their heartbeats during each interval without performing any manipulation to facilitate perceiving their heartbeats (e.g., taking the radial pulse). A signal tone at the beginning and end of each interval indicated its duration. After each interval, participants entered the number of heartbeats they had perceived. IAcc in the HCT was calculated according to the following formula:


$${IAcc}_{HCT}=\frac{1}{6}\sum_{k=1}^{6}1-\frac{\left|recorded\;heartbeats-perceived\;heartbeats\right|}{recorded\;heartbeats}$$

In the HDT (30), participants were presented with a visual stimulus (orange dot) for 80 ms which appeared either with a latency of 230 ms (S + trials, synchronous) or 530 ms (S - trials, delayed) after their R-wave on the screen. A trial contained ten consecutive visual stimuli with the same latency (12, 20). After each trial, patients were asked to indicate whether the visual stimulus had appeared ‘synchronously’ or ‘delayed’ to their own heartbeat. The HDT started with 10 training trials after which participants completed 20 experimental trials. IAcc in the HDT was calculated as the d’ parameter from signal detection theory (36). For this, S + trials correctly judged as ‘synchronous’ were defined as ‘hits’ and S - trials which were falsely classified as ‘synchronous’ were defined as ‘false alarms’:


$${IAcc}_{HDT}=d^{\prime }=z_{hit\;rate}-z_{false\;alarm\;rate}$$

### 2.5 EEG and ECG data acquisition, reduction, and analysis

A 32-channel Ag/AgCI active electrode EEG-system with BrainAmp amplifiers (Brain Products GmbH, Gilching, Germany) was used to record EEG. Electrodes were placed according to the 10/20-system (37) and referenced to FCz. Ag/AgCI electrodes were used to record bipolar vertical and horizontal electrooculogram (EOG) as well as ECG (Einthoven lead II configuration). The ECG signal was processed online by an AccuSync 42 (AccuSync Medical Research Corp., Milford, CT, USA) to detect R-waves, which was necessary to operate the HDT. The accuracy in R-wave detection is >99.8% with a time delay below 3 ms. EOG electrodes were placed above and below the right eye (vertical EOG) and at the outer canthi of each eye (horizontal EOG). Data were recorded with a 0.0159 Hz high-pass filter (time constant 10 s; 6 dB/oct passive RC filter) and a 250 Hz low-pass filter (30 dB/oct Butterworth) and digitized at 5 kHz. Data were down-sampled online at 1 kHz with a 450 Hz antialiasing filter (24 dB/oct Butterworth). The analysis of psychophysiological data was performed with BrainVision Analyzer, Version 2.20 (Brain Products GmbH, Gilching, Germany).

EEG data were re-sampled at 250 Hz, re-referenced to averaged mastoids (TP9-TP10) and bandpass filtered at 0.1–35 Hz (half power, 24 db/octave). Furthermore, a 50 Hz notch filter was applied to reduce line noise, which was present as EEG data acquisition was performed in an unshielded room at the hospital (38). Next, large non-stereotypical artifacts were excluded from further analyses. After excluding one faulty electrode of five participants each, eye blinks and saccades were corrected with the Restricted Infomax Independent Component Analysis (ICA) algorithm (39) in semiautomatic mode. Subsequently, R-waves were automatically detected and manually confirmed in the ECG data. EEG data were segmented into epochs ranging from 200 ms before to 1000 ms after the R-wave and baseline corrected (-200 ms to 0 ms).

HEP amplitudes were calculated as mean amplitudes in the time interval of 455–595 ms after the R-wave (‘normal’ HEP interval) (26, 40, 41) where the impact of the electro-cardiac field is considered minimal (42). Additionally, we extracted mean amplitudes for two control time intervals of the same length: an early control interval (180 to 320 ms) and a late control interval (860 to 1000 ms) (26, 41, 43). To examine gross effects of scalp location (20, 44, 45), electrodes (excluding reference channels) were pooled into 3x3 scalp sectors: anterior left (Fp1, F3/7, FC1/5), anterior central (Fz, FCz), anterior right (Fp2, F4/8, FC2/6), central left (C3, T7), central (Cz), central right (C4, T8), posterior left (CP1/5, P3/7, PO9/1), posterior central (Pz, Oz) and posterior right (CP2/6, P4/8, PO10/02).

HR and HRV-indices were calculated from the down-sampled (250 Hz) and bandpass-filtered (0.1–35 Hz, 24db/octave) ECG signal recorded during the 5-min resting period using the ARTiiFACT software (46). Cubic spline interpolation was applied to correct artifacts in interbeat intervals (IBI). HR was directly derived from RR-interval series and served as an indicator for sympathetic activation. The spectral analysis of the RR-interval series was based on Fast Fourier Transformation (FFT). As frequency-domain measures of HRV, we calculated relative power as the percentage of total power (47) for the high-frequency (HF; 0.15-0.4 Hz) and the low-frequency band (LF; 0.04-0.15 Hz) (48), respectively. While HF HRV reflects parasympathetic activation, LF HRV is sensitive to both sympathetic and parasympathetic activity (47).

### 2.6 Statistical analyses

HEP amplitudes were evaluated using a 2 x 3 x 3 x 3 repeated-measures ANOVA, including the within-subject factors ‘condition’ (resting period; HCT), ‘interval’ (early; HEP, late), ‘lobe’ (anterior; central; posterior) and ‘laterality’ (left; central; right). Degrees of freedom were adjusted applying the Greenhouse-Geisser correction and *post-hoc* analyses were conducted performing Bonferroni-corrected paired t-tests. ΔHEP (HCT – resting period) during the ‘normal’ HEP interval at electrode Cz was calculated as an indicator for filter system activity (20). Hypotheses (1) and (2) were tested by conducting two separate moderated hierarchical multiple linear regression analyses with IAcc_HCT_ and IAcc_HDT_ as the respective criterion variables. In the first step, we evaluated the associations of parasympathetic and sympathetic arousal (stage l.) and filter system activity (stage II.) with IAcc (stage III.) by simultaneously entering HF HRV, LF HRV, HR and ΔHEP as predictors. In the second step we tested whether strength of filter system activity as indicated by ΔHEP moderated the association between parameters of sympathetic arousal and IAcc by simultaneously entering the interaction term of LF HRV and ΔHEP and the interaction term of HR and ΔHEP in the regression models. All predictor variables were standardized before entering them in the regression models. Results of a sensitivity power analysis conducted with G*Power (49) revealed that for this regression model with seven predictors, the achieved sensitivity was *R^2^* = .17 (α = .05; 1 – β = .80). This rendered it possible to detect *R^2^* values of .24 previously reported in a study examining the predictive value of different cardiovascular parameters for IAcc (50). In follow-up analyses, we included depressive symptoms and anxiety as covariates into the first step of the regression models. The relationship between IAcc and pain intensity (hypothesis 3) was evaluated by calculating Pearson correlations between indicators of IAcc, i.e., IAcc_HCT_ and IAcc_HDT_, current pain intensity at session 2 and pain intensity in the past seven days. Single missing data on the depressive symptoms and anxiety domains of the PROMIS-29 v2.1 Profile were handled by response pattern scoring, the scoring method provided by the HealthMeasures Scoring Service (https://www.assessmentcenter.net/ac_scoringservice). For one patient, data on the pain intensity scale and the depressive symptoms and anxiety domains of the PROMIS-29 v2.1 Profile were missing completely. Further, due to equipment failure, data on the HDT were missing for two patients. Analyses involving these measures were thus performed excluding these patients (n= 74). For all analyses critical alpha level was set to *p* <.05 and data were analyzed using IBM SPSS Statistics (Version 28) predictive analytics software.

## 3 Results

### 3.1 Sample characteristics

The 76 participants of this study (69.7% females, *n* = 53) were between 21 to 65 years old (*M* = 50.7, *SD* = 9.2) and reported a mean pain duration of 10.3 years (range: 0.5 – 46.0 years). As primary diagnosis regarding their pain condition, 90.8% of the patients had a condition involving the musculoskeletal system and connective tissue, and 9.2% had a somatoform disorder. Most patients received analgesics (90.8%) with nonsteroidal anti-inflammatory drugs (63.2%), metamizole (36.8%), and opioids (15.8%) being the most frequently prescribed pain relievers. The most common comorbid diagnoses were hypertension (32.9%), disorders of the thyroid glands (25.0%), depressive disorders (23.7%), diabetes mellitus (11.8%) and anxiety disorders (10.5%). In addition to analgesics, most patients (80.3%) took other prescribed medication such as metabolic and endocrine drugs (50.0%), psychotropic drugs (25.5%) and drugs targeting the cardiovascular system (35.5%). **Table 1** provides an overview of further sample characteristics.

**Table 1:** Sample characteristics.

Variable	*M (SD)*	2	3	4	5	6	7	8	9	10	11	12	13
1 Age (years)	50.7 (9.2)	.25†	-.13	.02	-.14	-.10	-.13	-.15	-.08	-.16	.18	.19	.07
2 BMI (kg/m^2^)	28.2 (6.1)		-.07	-.14	-.02	.13	-.11	-.21	.07	-.16	.14	.18	.06
3 HR (bpm)	70.7 (9.0)			.03	-.09	-.12	.05	-.11	-.10	.08	-.08	.06	.10
4 LF (%)	38.4 (18.8)				-.65*	-.20	.08	-.14	-.16	.07	-.23†	-.15	-.16
5 HF (%)	29.5 (19.2)					.15	.15	.34*	.08	.08	.20	.08	.15
6 ΔHEP (μV)	0.07 (0.87)						.18	.06	-.08	.05	-.17	.01	.16
7 IAcc_HCT_	0.56 (0.22)							.24†	.03	.26†	-.17	-.13	.18
8 IAcc_HDT_	0.37 (1.03)								-.05	-.02	.02	-.13	-.08
PROMIS-29 v2.1													
9 Depression	55.4 (8.5)									.72*	.41*	.29†	.19
10 Anxiety	58.2 (8.3)										.12	.32*	.30*
11 Pain intensity	6.7 (1.7)											.40*	-.03
12 Current pain intensity	4.3 (2.1)												.18
13 Pain duration	10.3 (10.7)												
	n (%)												
CPG													
Grade l	2 (2.6)												
Grade ll	12 (15.8)												
Grade lll	19 (25.0)												
Grade lV	43 (56.6)												

*†p* <.05, **p <.01 (2-tailed); BMI, body mass index; HR, heart rate; LF, low frequency heart rate variability; HF, high frequency heart rate variability; ΔHEP, difference of heartbeat-evoked potentials heartbeat counting task – rest; IAcc_HCT_, interoceptive accuracy in heartbeat counting task; IAcc_HDT_, interoceptive accuracy in heartbeat detection task; CPG, Chronic Pain Grade.

### 3.2 Heartbeat-evoked potentials

**Figure 1:**
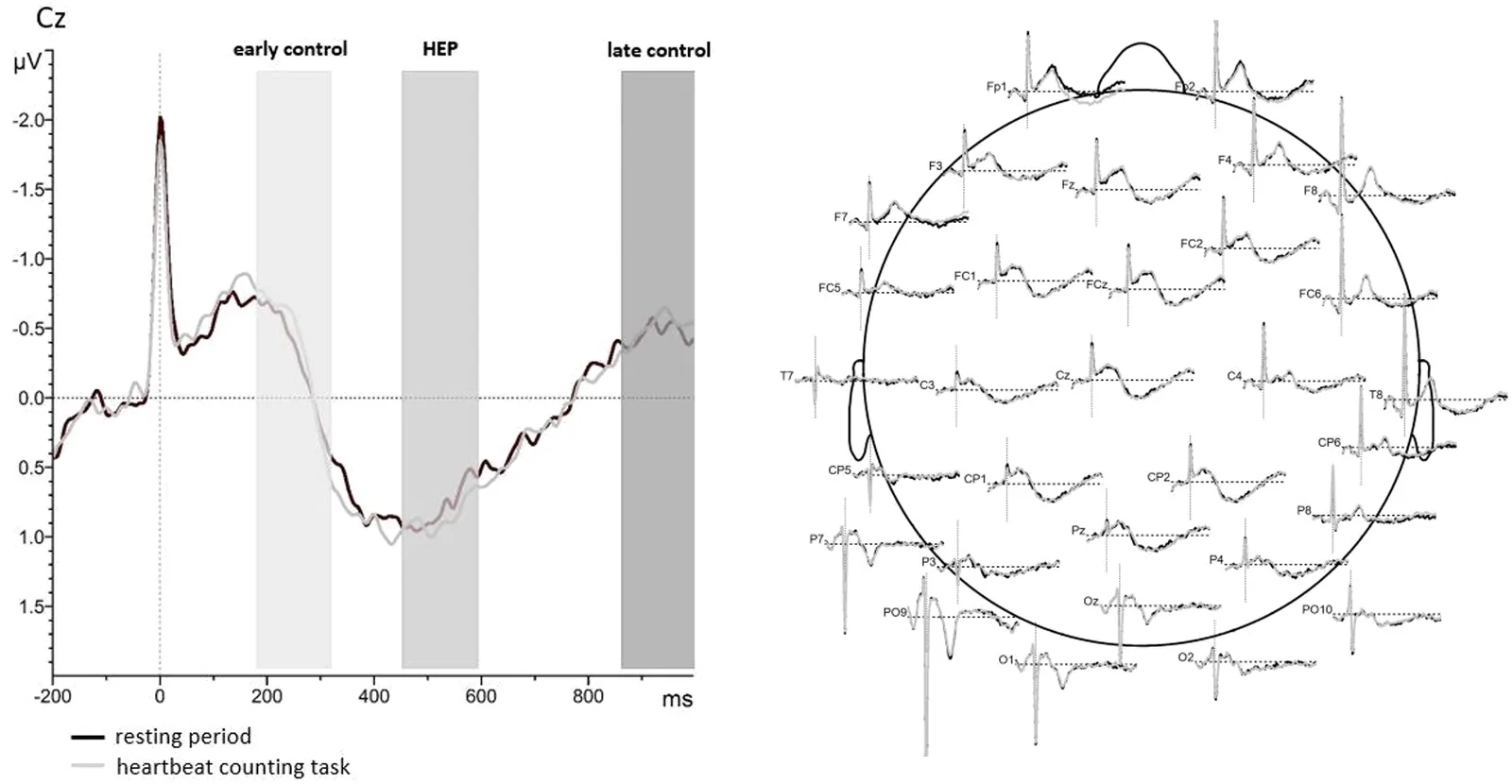
Heartbeat-evoked potentials (HEPs) did not differ between the resting period and the heartbeat counting task (HCT) in the ‘normal’ HEP interval.

**Figure 2:**
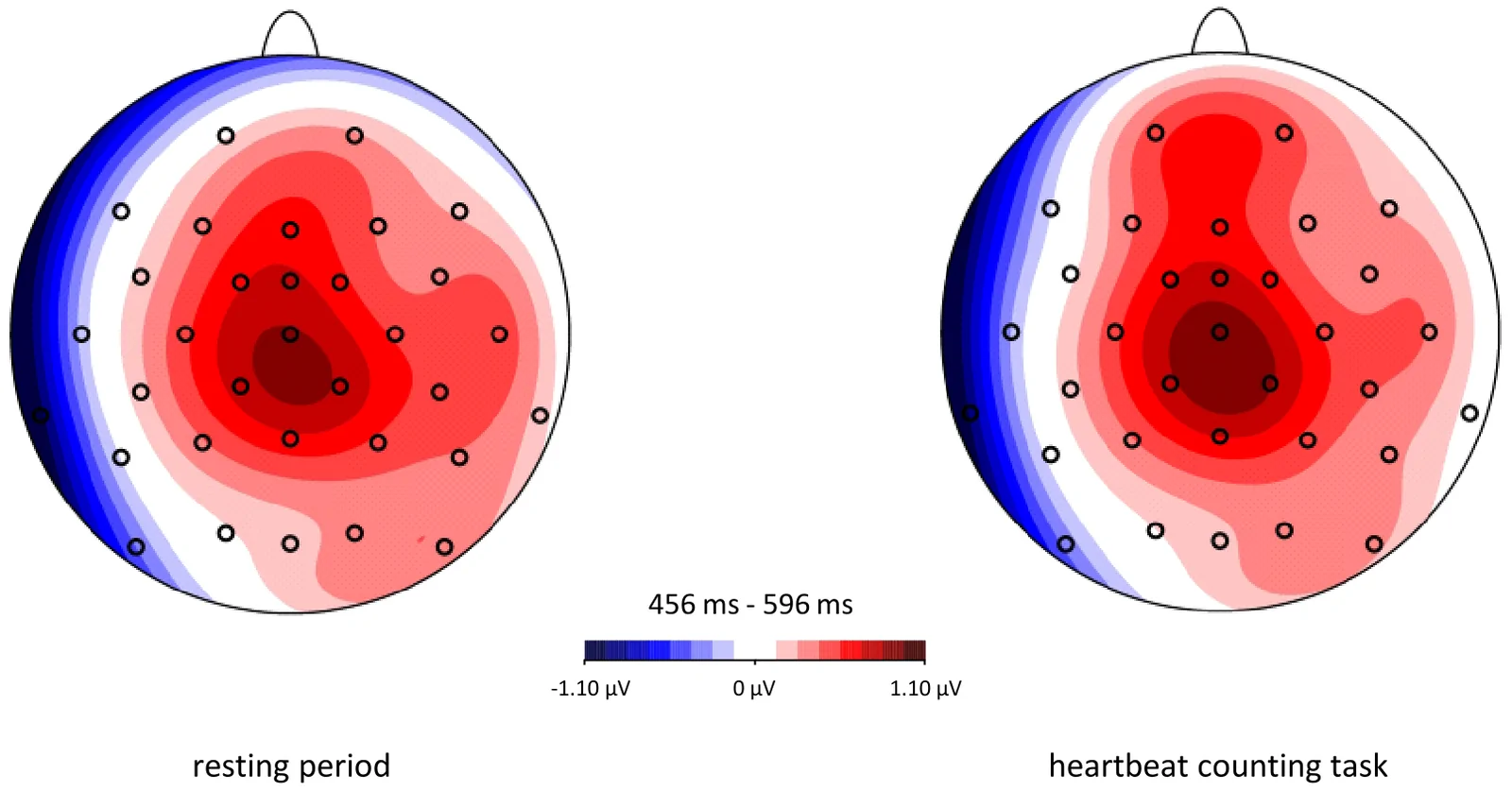
Scalp topographies of the heartbeat-evoked potentials (HEPs) for the ‘normal’ HEP interval during the resting period (left) and the heartbeat counting task (HCT; right).

HEP amplitudes did not differ between the resting condition and while performing the HCT (**Figure 1**). There was a significant three-way interaction ‘interval’ x ‘lobe’ x ‘laterality’, *F* (84.69, 351.41) = 8.25, *p* <.001, $\eta_{p}^{2}=0.099$. In the ‘normal’ HEP interval, HEP amplitudes were significantly higher over anterior central (*t* (75) = 3.17; *p* = .007; *d* = 0.363) and central electrode sites (*t* (75) = 4.45; *p* <.001; *d* = 0.511) than over the posterior central electrode sites. Additionally, HEP amplitudes in the ‘normal’ HEP interval were significantly higher at central right electrode sites than at anterior right electrode sites (*t* (75) = 2.66; *p* = .03; *d* = 0.305). These topographic differences could not be observed for the ‘early’ and ‘late’ control intervals. Scalp topographies for the ‘normal’ HEP interval during the resting condition and during the HCT are displayed in **Figure 2**.

### 3.3 Associations between stages l., ll., and lll

The results of the regression analyses are presented in **Table 2**. Concerning the prediction of IAcc_HCT_, the overall regression model was not significant at step 1 and step 2. HF HRV as well as LF HRV were the only significant predictors and both had a positive relationship with IAcc_HCT_. Neither HR and ΔHEP nor the interaction terms were significantly associated with IAcc_HCT_. When including depressive symptoms and anxiety as covariates into the regression analysis predicting IAcc_HCT_, HF HRV (step 1: β = .07, *p* = .036) and LF HRV (step 1: β = .07, *p* = .043) remained significant positive predictors of IAcc_HCT_. Further, anxiety was significantly and positively related to IAcc_HCT_ (step 1: β = .09, *p* = .020). Depressive symptoms, HR, ΔHEP and the interaction terms were not significantly associated with IAcc_HCT_. The overall model including the covariates was significant at step 1 (*R^2^* = .21, *F*(6, 68) = 2.98, *p* = .012) and step 2 (*R^2^* = .21, *F*(8, 66) = 2.18, *p* = .040).

**Table 2:** Moderated hierarchical regression models examining the associations between stages I, II and III.

Variable	Model I								Model II							
	IAcc_HCT_								IAcc_HDT_							
	β	*t*	*p*	*ΔR^2^*	*R^2^*	*F*	df	*p*	β	*t*	*p*	*ΔR^2^*	*R^2^*	*F*	df	*p*
Step 1				.12	.12	2.47	4, 71	.052				.14	.14	2.71	4, 69	.037
Constant	.56	22.67	<.001						.37	3.22	.002					
HF	.08	2.40	.019						.44	2.90	.005					
LF	.08	2.35	.022						.15	0.96	.339					
HR	.02	0.84	.405						-.08	-0.66	.509					
ΔHEP	.05	1.83	.071						.02	0.15	.879					
Step 2				.00	.12	1.61	6, 69	.158				.02	.15	2.01	6, 67	.076
Constant	.56	21.80	<.001						.39	3.30	.002					
HF	.08	2.27	.026						.41	2.60	.011					
LF	.08	2.23	.029						.14	0.86	.396					
HR	.02	0.84	.405						-.06	-0.55	.585					
ΔHEP	.05	1.70	.093						.02	0.19	.848					
ΔHEP x LF	.00	-0.09	.928						-.02	-0.18	.857					
ΔHEP x HR	.01	0.18	.855						.23	1.15	.253					

IAcc_HCT_ , interoceptive accuracy in heartbeat counting task; IAcc_HDT_, interoceptive accuracy in heartbeat detection task; HR, heart rate; LF, low frequency heart rate variability; HF, high frequency heart rate variability; ΔHEP, difference of heartbeat-evoked potentials heartbeat counting task – rest.

With regards to the prediction of IAcc_HDT_ the overall regression model was significant at step 1 but was no longer significant when the interaction terms were added in step 2. HF HRV was the only significant, positive predictor of IAcc_HDT_. LF HRV, HR, ΔHEP and the interaction terms were not significantly associated with IAcc_HDT_. HF HRV remained significantly and positively related to IAcc_HDT_ when adding depressive symptoms and anxiety as covariates (step 1: β = .43, *p* = .008). Neither depressive symptoms, anxiety, LF HRV, HR, ΔHEP nor the interaction terms predicted IAcc_HDT_. The overall regression model including the covariates was not significant at step 1 (*R^2^* = .14, *F*(6, 66) = 1.83, *p* = .107) or step 2 (*R^2^* = .15, *F*(8, 64) = 1.46, *p* = .189).

### 3.4 Associations between IAcc and pain intensity

Current pain intensity at session 2 was neither related to IAcc_HCT_ (*r* = -.13, *p* = .257) nor to IAcc_HDT_ (*r* = -.13, *p* = .262). Further, pain intensity in the past seven days was unrelated with either IAcc_HCT_ (*r* = -.17, *p* = .145) or IAcc_HDT_ (*r* = .02, *p* = .877).

## 4 Discussion

The current study investigated whether alterations in three components of processing of bodily signals, as posited by the perception-filter model can be observed in a sample of patients with chronic pain. Concerning the association between afferent bodily signal strength (stage I.) and IAcc (stage III.; hypothesis 1), we found that while HR did not predict either indicator of IAcc, LF HRV was a positive predictor of IAcc_HCT_. Further, HF HRV positively predicted both IAcc_HCT_ and IAcc_HDT_. With regards to the filter system activity (stage II.), HEP amplitudes did not differ between the HCT and the resting period. Moreover, strength of filter system activity, operationalized as the difference score in HEP amplitudes between these conditions, did not moderate any of the relationships between parameters of sympathetic arousal and indicators of IAcc (hypothesis 2). Neither indicator of IAcc (stage III.) was significantly associated with measures of symptom report, i.e., intensity of pain (hypothesis 3).

In contrast to our expectations, parasympathetic arousal, as indexed by HF HRV, was a positive predictor of both indicators of IAcc. Furthermore, also LF HRV was identified as a positive predictor of IAcc_HCT_, but no other association between parameters of sympathetic arousal and indicators of IAcc reached statistical significance. One possible conclusion could be that sympathetic arousal, potentially affecting cardiac signal strength, is not a consistent predictor of cardiac IAcc. Alternatively, considering that LF HRV is sensitive to sympathetic and parasympathetic changes (47), the positive associations of both HF and LF HRV with indicators of IAcc might imply that parasympathetic activation plays an important role in IAcc. This is in line with findings of prior research investigating this relationship (51–53) and might shed light on fundamental mechanisms of cardiac signal properties underlying cardiac interoception. Previous studies demonstrated that stroke volume, cardiac contractility and sympathetic activation facilitating cardiac inotropy enhance IAcc (50, 54, 55). This may be because with increasing contractility, heartbeats can be discriminated more easily from background noise, i.e., cardiac relaxation during diastole. For the same reason, opposite effects may be observed when focusing on chronotropic indicators of (para-)sympathetic tone, such as HR and HRV, as higher HR and lower HF HRV are potentially associated with lower stroke volume and shorter diastolic periods, making the differentiation between signal (i.e., heartbeat) and background noise more difficult. Future studies on chronic pain patients should investigate whether assumptions of the perception-filter model can be confirmed if indicators of contractility and inotropic activation are included.

The topography of the HEP amplitudes was comparable to previous studies, with a positivity shift in the ‘normal’ HEP interval and highest amplitudes over Cz. In contrast to previous studies investigating the perception-filter model in high symptom reporters and patients with somatoform disorders (20, 21), however, we observed comparable HEP amplitudes in the HCT and the resting period. This finding suggests the absence of attentional effects on HEP amplitudes (28) and - in accordance with the perception-filter model - suggests decreased filter system activity in patients with chronic pain. Nevertheless, strength of filter system activity did not moderate the relationship between signal strength and IAcc. Considering that the current study only included patients receiving inpatient treatment at a specialized tertiary hospital, this finding could be attributed to the composition of the sample. On average, participants had been experiencing pain for about 10 years, and the majority (81.6%) graded their pain as highly disabling (32). It could be argued that at this stage of disease progression dysfunction of the filter system was pronounced for most of the patients in the sample, leaving not enough variance in the strength of filter system activity to exert a moderating effect. Future, large-scaled studies including a broad range of chronic pain patients with different degrees of impairment are, therefore, needed to elucidate the role of filter system activity in the perception of physical symptoms in chronic pain.

Based on the perception-filter model, positing enhanced perception of afferent physical signals, we predicted a positive association between IAcc and symptom report. Nevertheless, indicators of IAcc and pain intensity were not significantly correlated in the current study. Considering that the decreased capacity of the filter system might result in the amplification of all afferent bodily signals, it is possible that amplified physical signals from other modalities, notably pain, interfered with the processing of cardiac signals. This is in line with the notion that an individual’s capacity to process information at a given time is limited and signals, therefore, compete for attention as put forward by Pennebaker’s competition of cues model (56). In light of the attention demanding characteristic of pain (57) this would imply that in relation to the sensation of pain the perceptual intensity of heartbeats might have been reduced among the patients of the current study. Indeed, except for the correlation between pain intensity in the past seven days and IAcc_HDT_, we found a small, non-significant trend toward a negative association between pain intensity and IAcc. In line with this interpretation, a systematic review on interoceptive alterations in chronic pain conditions (58) showed that cardiac IAcc is inversely related to symptom severity. Accordingly, the current findings challenge the assumption of a generally increased perception and detection of afferent internal bodily signals across different modalities in chronic pain. Alterations in interoceptive signal processing as described in the perception-filter model may, therefore, only apply to symptom-specific sensory input. Increased IAcc in chronic pain patients would thus only be expected concerning physical signals originating from the region of their symptoms. Nevertheless, the extant literature is inconclusive and has mainly focused on the perception of external, symptom-related signals. While several studies have found perceptual amplification of and increased sensitivity to external noxious stimuli in patients with persistent pain (59–61), others did not show enhanced perception of symptom-related sensations, such as muscle tension (62–64). To further disentangle the role of interoception in the etiology of chronic pain, future studies should focus on developing symptom-specific paradigms which render it possible to meaningfully assess interoceptive alterations in the pain system.

As depression and anxiety are common comorbidities of chronic pain (2) and have been found to be associated with alterations in IAcc (11) we included both as control variables in the regression models. This did not affect the magnitude of the associations found between the different stages of the perception-filter model, suggesting that differences in depressive symptoms and anxiety did not affect the findings of this study. In line with results of previous studies (65, 66), anxiety was, however, a positive predictor of IAcc_HCT_ underscoring the importance of paying attention to the role of this comorbidity when investigating interoception in chronic pain.

### 4.1 Limitations

(1) The majority of participants included in the current study had a multimorbid disease profile and received a broad range of different medications. While such a complex pattern of comorbidity is common in this group of patients (67, 68), and thus increased the ecological validity of the study, it rendered it impossible to evaluate specific effects of medical diagnoses or medication on the constructs assessed. We thus cannot rule out that comorbidity or medication impacted the current results. (2) We investigated hypotheses on bodily signal processing in chronic pain derived from the perception-filter model only with regards to cardiac interoception, as this modality is currently the only one that allows for the assessment of all stages of the model. It is unclear whether the findings of the current study can be transferred to the processing of physical signals of other interoceptive domains, such as the pain system. Hence, the current study did not test the perception-filter model as such, but rather specific hypotheses derived from the model with regards to cardiac indices, interoceptive accuracy, and symptom report in chronic pain. (3) As HF power is also sensitive to other factors of influence such as respiration rate and volume (47, 69), it cannot be ruled out that the variance in HF HRV was partially affected by these indicators, which were not assessed in this study. Hence, caution is needed when interpreting the physiological origin of HF HRV. This is particularly important as lower HRV in individuals with high levels of MUS might be due to higher respiration rates (20). Nevertheless, it should be emphasized that even if HF HRV varies with respiratory changes, these changes still result in variations in cardiac signal strength due to central (para-)sympathetic tone. Furthermore, it needs to be acknowledged that HRV measures are not ‘pure’ measures of cardiac signal strength, but always reflect regulatory circuitries of afferent and efferent cardiac signals. Finally, additional factors affecting HRV, such as physical fitness, might have contributed to the findings of the current study. (4) Due to the cross-sectional design of the study, conclusions about cause-and-effect relationships cannot be drawn. (5) There is an ongoing debate in the literature whether the HCT employed in the current study to assess IAcc, not only reflects cardiac interoception, but also non-interoceptive factors, such as knowledge of one’s own heart rate and the ability to estimate the duration of time intervals. Although we followed all recommendations to mitigate potential effects of these non-interoceptive factors, such as instructions only to count actually perceived heartbeats (70), IAcc_HCT_ scores might not be a ‘pure’ measure of stage (III.): perception. In contrast to previous studies (71, 72), which recommended open eyes during the HCT to control for the visual input during the task, participants in our study did not receive any instructions to open or close their eyes. Although there are no studies showing systematic differences between both conditions, inter-individual differences may have contributed to variability in IAcc_HCT_ in the current study. (6) The high potential burden of chronic pain conditions in the current study resulted in high levels of pain killer intake, including opioids and psychotropic medication. These substances might have affected both cardiac activity (e.g., stronger parasympathetic tone) and the perception of cardiac signals (e.g., reduced difficulties to focus attention on heartbeats). Nevertheless, the heterogeneity of substances and the moderate sample size limited the possibility to statistical control for medication intake.

### 4.2 Conclusions

Focusing on the processing of cardiac signals, the current results showed that alterations in the processing of bodily signals, as previously posited for MUS, do not apply to chronic pain conditions. While chronic pain patients seem to be characterized by reduced filter system activity, the associations between the three stages of afferent bodily signal processing was not confirmed. In light of the high disease burden of chronic pain, an in-depth understanding of mechanisms underlying the pathogenesis of persistent pain is essential. Future studies should, therefore, investigate if deficiencies in the interoceptive filter system is limited to cardiac signals or generalizes to other interoceptive signals, and if this dysfunction might be overcome by novel intervention approaches. This research will help to establish the clinical relevance of interoceptive alterations for chronic pain.

## Data Availability

The participants of this study did not give written consent for their data to be shared publicly. Therefore, in order to protect the personal data of the participants in accordance with the European General Data Protection Regulation, the data supporting this study cannot not be disclosed. Requests to access the datasets should be directed to mirjam_thomas@web.de.
